# Evidence‐based prescription of corticosteroids for COVID‐19 patients: A comparison between general medicine and other specialty in Japan

**DOI:** 10.1002/jgf2.602

**Published:** 2022-12-30

**Authors:** Kota Sakaguchi, Takashi Watari, Yasuharu Tokuda

**Affiliations:** ^1^ General Medicine Center Shimane University Hospital Shimane Japan; ^2^ Medicine Service VA Ann Arbor Healthcare System Ann Arbor Michigan USA; ^3^ Department of Medicine University of Michigan Medical School Ann Arbor Michigan USA; ^4^ Muribushi Okinawa Clinical Training Center Okinawa Japan; ^5^ Tokyo Foundation for Policy Research Tokyo Japan

**Keywords:** COVID‐19, glucocorticoids, pandemic, pharmacoepidemiology, prescription

## Abstract

**Background:**

The difference of evidence‐based prescription for COVID‐19 patients between general medicine and other specialty in Japan is unclear.

**Methods:**

A retrospective observational study using a hospital‐based administrative database was conducted for the differences between departments for prescriptions of glucocorticoids in COVID‐19 patients.

**Results:**

Of 65,156 patients, 23,348 met indication for glucocorticoids. General medicine physicians in Japan were more likely to prescribe corticosteroids for COVID‐19 patients than other departments after its evidence was published (adjusted odds ratio, 1.32; *p* < 0.001; 95% confidence interval, 1.11–1.56).

**Conclusions:**

General medicine physicians were more likely to practice evidence‐based care for COVID‐19 patients.

## Abstract 2

This study aimed to compare glucocorticoid prescriptions issued by different medical departments in Japan, during the COVID‐19 pandemic.

## 1 INTRODUCTION

It is crucial to determine the quality of general care for COVID‐19 patients to assess patient outcomes. In several countries, general practitioners had to contribute as the mainstay of outpatient and inpatient care because infectious disease specialists and respiratory physicians could not treat all patients during the COVID‐19 pandemic.^1^, ^2^ Numerous clinical trials have been conducted to date, with the aim of establishing an effective treatment. In particular, the report of an absolute risk reduction in 28‐day mortality by administering dexamethasone to COVID‐19 hospitalized patients requiring oxygen therapy and mechanical ventilation on June 16, 2020, was a breakthrough.^3^ In the United Kingdom, based on this scientific evidence, the administration rate of corticosteroids to eligible patients increased from 27.5% in the week before June 16, 2020, to 75%–80% in January 2021, and the successful implementation of new treatment methods with a high evidence level was recognized, mainly in general medicine departments.^4^

However, the circumstances surrounding the implementation of corticosteroids in COVID‐19 patients by general medicine departments in Japan are unclear. Potential inequalities in treatment according to specialty during pandemics are critical to individuals, communities, and nations. Therefore, we aimed to elucidate the quality of general care for COVID‐19 patients. We examined the past situation of glucocorticoid prescription for COVID‐19 patients after solid evidence had been published and whether there were differences by specialty in Japan during this pandemic.

## 2 METHODS

The current study used the largest nationwide hospital administrative database, called Diagnosis Procedure Combination (DPC) data in Japan, containing data from 25% (438 of 1750) of all acute care hospitals (DPC hospitals). The DPC dataset used in the current study was collected from the Ministry of Health, Welfare, and Labour of the government of Japan by Medical Data Vision (MDV), Co., Tokyo, Japan (as of April 1, 2021).^5^ In the dataset, the age and gender distribution of registered patients is comparable to the distribution of patients at healthcare institutions nationwide as published by the government of Japan, thus representing the national data.^1^ The current study collected the MDV DPC data from January 1, 2010 to November 30, 2021, and focused on patients of all ages with confirmed (RT‐PCR test positive) acute COVID‐19 (International Classification of Diseases and Related Health Problems, 10th Revision diagnosis code U071) who required admission, with acute COVID‐19 as the major cause for the admission. The data from the first admission were used in the current study.

### 2.1 Variables

The data used in this study included demographics, clinical characteristics, corticosteroid use, and departmental characteristics (general medicine, respiratory medicine, emergency medicine, internal medicine, pediatrics, cardiology, and gastroenterology) (Table 1). We followed the Japanese Medical Specialty Organization for specialty classification by the physicians.^6^

**TABLE 1:** The characteristics and comorbidities of patients with COVID‐19 in Japan

	June 16, 2020–November 30, 2021
Demographic and clinical characteristics	
Total no. of patients	23,348
Age, mean (SD), years	53.7 (0.08)
Age group, no. (%)	
>65	11,540 (49.3)
Gender, no. (%)	
Male	14,806 (63.4)
Female	8542 (36.6)
Comorbidities, no. (%)	
Congestive heart failure	3710 (15.9)
Acute myocardial infarction	297 (1.3)
Hypertension	10,075 (43.2)
Stroke	1120 (4.8)
Chronic kidney disease	1391 (6.0)
Diabetes	11,221 (48.1)
Obesity (BMI > 30)	5497 (23.5)
Chronic lung disease	6034 (25.8)
Rheumatologic arthritis	16 (0.1)
Smoking	10,905 (46.7)
Cancer	1341 (5.7)
ARDS	1179 (5.1)
Dementia	1846 (7.9)
ICU admission	2945 (12.6)
Mechanical ventilation	2393 (10.3)
Steroid use	14,763 (63.2)

*Note*: Data are presented as number (percentage) of patients unless otherwise indicated.

Abbreviations: ARDS, acute respiratory syndrome; BMI, body mass index; ICU, intensive care unit.

Standard descriptive statistics were used to calculate the number, percentage, mean, median, and interquartile range (IQR) for each piece of data. The chi‐square test or Fisher's exact test was used for comparisons of categorical data. For continuous variables, t‐tests or Wilcoxon rank‐sum tests were employed. Additionally, multivariate linear and logistic regression analyses were performed to examine the factors associated with cognitive bias. Variables were adjusted based on their clinical relevance and previous studies.^3^ The criteria used for the explanatory variables were factors associated with steroid prescriptions among COVID‐19 patients in previous studies^3^ and factors that were significant at *p* < 0.05, based on univariate screening and univariate regression. Specifically, general internal medicine was selected for the multiple logistic regression analysis of the presence of steroid prescriptions. All analyses were performed using the Stata statistical software (Stata Corp. 2015, Stata 17 Base Reference Manual). All tests were two tailed, and statistical significance was set at *p* < 0.05.This study was approved by the appropriate ethics committee.

## 3 RESULTS

Of the 65,184 COVID‐19 patients (female, 42.5%; mean age ± SD, 53.7 ± 21.6) included in this study, 23,348 (35.8%) received oxygen therapy, with dexamethasone indicated. In fact, 14,763 (63.2%) patients were prescribed dexamethasone, and the clinical characteristics of these patients included a high proportion of diabetes (11,221 patients [48.1%]) and smoking (10,905 patients [46.7%]) (Table 1). The general medicine department prescribed corticosteroids significantly more often than other departments (adjusted odds ratio, 1.27; *p* = 0.006; 95% CI, 1.07–1.51). Moreover, we found that diabetes (adjusted odds ratio, 1.32; 95% CI, 1.25–1.40) and mechanical ventilation (adjusted odds ratio, 1.08; 95% CI, 1.11–1.56) were factors in the patient background for high corticosteroid prescription (Table 2).

**TABLE 2:** Predictor variables and steroid use of patients with COVID‐19

Variables	Adjusted OR (95% CI)	*p* value
GIM	1.27 (1.07–1.51)	0.006
Age > 65 years	0.73 (0.69–0.78)	<0.001
Male sex	1.14 (1.08–1.21)	<0.001
Obesity (BMI > 30)	0.88 (0.81–0.95)	0.002
Smoking	0.92 (0.86–0.97)	0.002
Coma	0.46 (0.30–0.69)	<0.001
Shock	0.42 (0.07–2.55)	0.349
Cancer	0.92 (0.82–1.04)	0.203
Liver cirrhosis	1.10 (0.78–1.56)	0.579
Chronic lung disease	1.07 (0.10–1.14)	0.064
Diabetes	1.32 (1.25–1.40)	<0.001
Stroke	0.87 (0.75–1.01)	0.067
Heart failure	0.98 (0.90–1.07)	0.701
Chronic kidney disease	1.09 (0.96–1.24)	0.170
Dementia	0.54 (0.49–0.60)	<0.001
ICU	1.02 (0.93–1.13)	0.633
Mechanical ventilation	1.08 (0.97–1.19)	0.177

Abbreviations: CI, confidence interval; GIM, general internal medicine; OR, odds ratio.

## 4 DISCUSSION

This is the first study to analyze corticosteroid prescription and the department of admission for COVID‐19 patients using a large Japanese database. We found that the general medicine department was significantly more likely to use corticosteroids in COVID‐19 patients requiring oxygen therapy and mechanical ventilation at the time of admission than other departments. In addition to individual behavioral change, factors involved in decision‐making must be considered when implementing new therapeutic interventions, and in several cases, the complexity of the intervention and difficulty in reaching consensus with others have been identified as barriers.^7^ In previous studies, the Department of General Medicine collaborated with nurses and other frontline care team members during COVID‐19 treatment, which facilitated consensus building regarding new healthcare delivery methods and information sharing.^8^, ^9^ This may have been a factor in the rapid introduction of corticosteroids with a high level of evidence and decision‐making based on scientific evidence.

This study had several limitations. First, it only analyzed data from COVID‐19 patients in DPC‐registered hospitals in Japan. This is an inadequate sample size compared with the number of patients published by the MHLW.^10^ Second, because of the characteristics of the data described above, there may be insufficient assessment of the expertise of physicians involved in the prescription of corticosteroids.

In conclusion, we evaluated the characteristics of corticosteroid prescriptions for COVID‐19 patients after data on treatment with a high level of evidence was published and found that general medicine departments prescribed more corticosteroids for COVID‐19 patients than other departments. This was a result of the application of treatment by general medicine departments immediately after data with high level of evidence regarding the treatment were published.

## FUNDING INFORMATION

This study was supported by the national academic research grant funds [JSPS KAKENHI: 20H03913]. The sponsor of the study had no role in the study design, data collection, analysis, or preparation of the manuscript.

## ETHICAL APPROVAL

This study was conducted per the Declaration of Helsinki and the Strengthening the Reporting of Observational Studies in Epidemiology (STROBE) statement guidelines. This study was approved by the Institutional Ethics Committee of the Muribushi Okinawa Center for Teaching Hospitals (No. 2021‐9).

## PATIENT CONSENT STATEMENT

None.

## CLINICAL TRIAL REGISTRATION

None.

## CONFLICT OF INTEREST

The authors have stated explicitly that there are no conflicts of interest in connection with this article.

## Data Availability

The data that support the findings of this study are available from the corresponding author upon reasonable request.
